# A hierarchy of time scales supports unsupervised learning of behavioral sequences

**DOI:** 10.1186/1471-2202-16-S1-P78

**Published:** 2015-12-18

**Authors:** Samuel P Muscinelli, Wulfram Gerstner

**Affiliations:** 1 School of Life Sciences, Brain Mind Institute and School of Computer and Communication Sciences, École Polytechnique Fédérale de Lausanne, 1015 Lausanne, Switzerland

## 

Playing the piano, speaking or playing tennis are just few examples of behavioral situations in which we need to perform sequences of actions. The neurophysiological mechanisms that underlie the production and learning of such sequences are far from being understood and several crucial issues arise in biologically plausible models: first, there is a huge gap of time scales between the response of single neurons, which is on the order of tens of milliseconds, and common behavioral situations, which usually span several seconds. Moreover, behavioral sequences are often complex, i.e. they cannot be described as Markov chains and thus require to relate distant parts of the sequence. Finally, similar to its biological counterpart, a candidate model should be able to learn new sequences with biologically plausible learning rules.

We devise a simplified model of neural populations with the aim of producing slow sequences of neural activation to correlate with behavioral sequences. We exploit spike frequency adaptation of single neurons to introduce a slow process [1] in the population dynamics in order to fill the gap between different time scales described above. Our model features a hierarchy of time scales inspired by evidence of different time scales among different areas in the brain [2,3]. This allows a separation of neural coding into varying levels of temporal detail: the sequence produced in a "faster" area will be regulated by the activity of "slower" areas in a hierarchical fashion. This provides a mechanism to deal with non-Markovianity thanks to the longer memory capacity of "slower" areas. Finally, we show that it is possible to learn the appropriate inter-area synaptic connections using biologically plausible learning rules, exploiting the decrease in activity due to adaptation. This approach leads to the development of temporal receptive fields associated to subparts of the desired sequence with hierarchical levels of detail.

Our model constitutes a promising approach to temporal learning, showing how an appropriate neural substrate with a hierarchy of time scales can lead, even without any error or reward signal, to the learning of slow and complex sequences.

## References

[B1] Pozzorini C Naud R Mensi S Gerstner W Temporal whitening by power-law adaptation in neocortical neurons Nat Neurosci 2013 16 7 942 948 23749146 10.1038/nn.3431

[B2] Kiebel SJ Daunizeau J Friston KJ A hierarchy of time-scales and the brain PloS Comput Biol 2008 4 11 e1000209 19008936 10.1371/journal.pcbi.1000209 PMC2568860

[B3] Hasson U Yang E Vallines I Heeger D Rubin N A hierarchy of temporal receptive windows in human cortex J Neurosci 2008 28 10 2539 2550 18322098 10.1523/JNEUROSCI.5487-07.2008 PMC2556707

